# Practical Medicine

**Published:** 1879-09

**Authors:** 


*Collaborators:*

DR. H. GRADLE, DR. L. W. CASE, DR. R. PARK,
DR. R. TILLEY.

### PRACTICAL MEDICINE.

TUBERCULAR IRITIS.—(*LaFrance Médicale*), July 12, 1879. A patient was presented to the "Societé de Chirurgie," of Paris, by Mr. Parinaud suffering from primitive tuberculosis of the eye. There was but one opinion on the question of diagnosis. The patient was a child of twelve years, strumous; the father died five weeks previously, and the affection of the eye is referred to the same date.

The cornea presented a whitish and soft appearance over the whole inferior half. In the lower part of the iris was discernable a rose-colored tumor from which emanated other smaller tumors. In the upper part of the iris were two other tumors. By the aid of oblique illumination other little projections were noticeable, which resemble the granulations of tuberculosis, and situated near the border of the pupil. The lesions resemble hypopyon. The softening of the cornea only affected the posterior layers of the cornea. The anterior layers perfectly transparent. The fundus of the eye could not be seen which rendered the existence of deeper lesions probable. The disease displayed no marked activity.

The affected eye—the left—was wholly insensible to light. The right eye was normal. The general health was not perceptibly changed. It was not possible to discover the presence of tubercle in any other organ. No reason whatever to suspect inherited syphilis.

The question of treatment was what elicited the discussion. To prevent a general tuberculosis, as the affection was supposed to be confined to the eye, the enucleation of the ball was advocated by Messrs. Th. Auger, Giraud, Teulon, Le Fort. Mr. Verneuil and others opposed the idea of enucleation from the fact that cretification sometimes takes place in tubercular deposits, and from the possibility of an operation exciting the disease to greater activity.

